# Impact of orthodontic-induced facial morphology changes on aesthetic evaluation: a retrospective study

**DOI:** 10.1186/s12903-023-03776-4

**Published:** 2024-01-05

**Authors:** Chao Liu, Siyuan Du, Zhengliang Wang, Shikai Guo, Mengjuan Cui, Qianglan Zhai, Manfei Zhang, Bing Fang

**Affiliations:** 1 Department of Orthodontics, Jiao Tong University School of Medicine & Shanghai Key Laboratory of Stomatology &, Shanghai Ninth People’s Hospital, Shanghai Research Institute of Stomatology & National Clinical Research Center for Oral Diseases, Shanghai, 200011 China; 2 Bio-X Institutes, Key Laboratory for the Genetics of Developmental and Neuropsychiatric Disorders, Ministry of Education, Shanghai Jiao Tong University, Shanghai, 200030 China; 3 Department of Plastic Surgery, Xi’ an International Medical Center Hospital, Xi’ an City, 710000 Shaanxi Province China

**Keywords:** Orthodontic treatment, Facial aesthetics, Phenotypic grouping, Aesthetic evaluations, Facial landmarks

## Abstract

**Background:**

The profound influence of orthodontic treatments on facial aesthetics has been a topic of increasing interest. This study delves into the intricate interplay between orthodontic treatments, facial feature alterations, and aesthetic perceptions.

**Methods:**

A total of 73 patients who had undergone orthodontic treatment were included in this study. Facial photographs were taken before and after treatment. Ten orthodontists provided facial aesthetic ratings (FAR) for each patient's frontal, profile, and overall views. 48 facial landmarks were manually placed by the orthodontists and normalized using Generalized Procrustes analysis (GPA). Two types of phenotypes were derived from facial landmarks. Global facial phenotypes were then extracted using principal component analysis (PCA). Additionally, 37 clinical features related to aesthetics and orthodontics were extracted. The association between facial features and changes in FAR after orthodontic treatment was determined using these two types of phenotypes.

**Results:**

The FAR exhibited a high correlation among orthodontic experts, particularly in the profile view. The FAR increased after orthodontic treatment, especially in profile views. Extraction of premolars and orthognathic surgery were found to result in higher FAR change. For global facial phenotypes, the most noticeable changes in the frontal and profile views associated with FAR occurred in the lip area, characterized by inward retraction of the lips and slight chin protrusion in the profile view, as well as a decrease in lip height in the frontal view. The changes observed in the profile view were statistically more significant than those in the frontal view. These facial changes were consistent with the changes from orthodontic treatment. For clinical features, two profile features, namely pg.sm.hori and pg.n.ls, were found to be associated with FAR following orthodontic treatment. The highest FAR scores were achieved when pg.sm.hori was at 80° and pg.n.ls was at 8°. On the other hand, frontal clinical features had a subtle effect on FAR during orthodontic treatment.

**Conclusions:**

This study demonstrated that orthodontic treatment improves facial aesthetics, particularly at lip aera in the profile view. Profile clinical features, such as pg.sm.hori and pg.n.ls, are essential in orthodontic treatment which could increase facial aesthetics.

**Supplementary Information:**

The online version contains supplementary material available at 10.1186/s12903-023-03776-4.

## Background

Facial aesthetics are always of general interest. One of the important reasons patients seek orthodontic care is to improve their facial attractiveness. Orthodontic treatment targets the dentition and the maxillomandibular relationships to create a considerable impact on facial esthetics. Orthodontic tooth movement and alveolar bone remodeling can cause facial morphological changes, which are closely related to aesthetic perception, as they interact and collectively determine an individual's facial aesthetic evaluation [1, 2]. Previous research findings indicate that the changes of lip position after orthodontic treatment (Ls-SnPog', Li-SnPog', and Li-PrnPog') exhibited notable quadratic correlations with the assessment of facial attractiveness in both frontal and lateral profiles [3, 4]. Furthermore, a study has revealed that the mentolabial sulcus angle increased in the incisor tipping group, whereas it decreased in the incisor translation group, leading to variations in facial aesthetics [5]. However, previous studies primarily focused on the changes in profile and the lower third of the face, encompassing alterations in teeth and jawbone resulting from orthodontic treatment [1, 2, 6–8], while studies investigating overall facial aesthetic changes have been relatively scarce. In clinical practice, it has been observed that improvements in the profile do not always correlate positively with overall facial aesthetics. Some patients, despite undergoing orthodontic treatment that altered their facial profile through means such as tooth extraction and orthodontic correction, did not experience significant enhancement in overall facial aesthetics, and even encountered aesthetic losses [7, 9]. Therefore, studies based on overall facial aesthetics are urgently needed.

Despite some studies have been analyzing facial photographs since 1933 [10], cephalometric analysis is still an important basis for developing orthodontic treatment [11]. Comprehensive assessment of facial aesthetics requires consideration not only of changes in dental and skeletal parameters but also the integration of the patient's overall facial morphology and baseline characteristics [7]. From an aesthetic point of view, facial soft tissues are more judgmental. However, there is a lack of both methods and metrics for soft tissue measurement and analysis relative to cephalometric measurements. The more commonly used clinically are still E-line [12], angle of facial convexity [13], and nasolabial angle [14]. With the rocketing progress of smart devices in recent years, facial morphological changes before and after orthodontic treatment can be easily evaluated even by smartphone-based facial scanning that could be a viable tool for facially driven orthodontics [15]. Hence, the study of measurement and analysis of facial photographs can help in designing valuable analytical tools.

The aim of this study was to investigate the impact of orthodontic treatment induced changes in facial morphology on facial aesthetics enhancement and analyze the key factors involved, which is crucial for optimizing treatment plans. In this study, we utilized pre- and post-orthodontic treatment photographs of patients, combined with expert evaluations, to assess facial aesthetics from the frontal and profile views, as well as overall assessment. We identified two important clinical facial features in orthodontic treatment that are associated with facial aesthetics. Our comprehensive understanding of the aesthetic outcomes related to orthodontic treatment can assist in optimizing treatment planning, enhancing patient satisfaction, and advancing the field of orthodontics and providing more accurate and comprehensive guidance for clinical practice and aesthetic evaluation.

## Methods

### Study design and sample recruitment

This study is retrospective research. A total of 73 eligible patients were recruited from the Department of Orthodontics, Ninth People's Hospital, Shanghai Jiao Tong University School of Medicine, who underwent orthodontic treatment between January 1st, 2019, and December 31st, 2020. For each participant, we collected frontal, 45-degree left and right, and 90-degree profile facial photographs before and after orthodontic treatment.

Inclusion criteria encompassed:

- (1) Participants of any gender who had undergone at least 2 years of orthodontic treatment and possessed complete data information.
- (2) Participants underwent orthodontic treatment throughout the entire process under the supervision of the Chief Physician, which represents a relatively higher level of orthodontic treatment expertise.

Exclusion criteria comprised:

(1) Participants who had undergone plastic surgery during the interval between the two orthodontic photographs or (2) had a history of maxillofacial trauma during this period.

This study received ethical approval from the Ethics Committee of Shanghai Ninth People's Hospital, Shanghai Jiao Tong University School of Medicine (Approval No.: SH9H-2021-TK461-1) and was registered with the Chinese Clinical Trial Registry (Registration No.: CTR2100050216).

### Facial photographs processing

All facial photographs were taken under standard conditions for treatment comparison. When taking facial photos, the patient should sit upright with both eyes looking straight ahead, the head parallel to the Frankfort horizontal plane (natural head position), habitual occlusion, and relaxed lips and facial muscles. For capturing frontal images: the camera should be strictly positioned at a fixed distance and location, aligned with the horizontal plane passing through the orbits and ears, and facing the midline of the face. For capturing profile images: the camera should be aligned with the ear canal, ensuring that the ears are not covered by hair. To facilitate a comprehensive evaluation of facial morphology, in addition to the aforementioned frontal and profile images, it is common to include photographs of the patient smiling and semi-profile (45°) images.

Due to the potential influence of the patient's clothing on subsequent evaluations, for photograph at each angle of the sample, we only cropped and retained the facial region to minimize the cofounding factors of clothing. We used the trichion point (tri) as the highest point, the gnathion point (gn) as the lowest point, and the contours of the face on both sides as the cropping boundaries for the left and right sides.

For each sample, we retained the frontal view photographs (Supplementary Fig. 1a), 90° right profile view photographs (Supplementary Fig. 1b), and overall view photographs (the combination of 90° right, 45° right, frontal, 45° left, and 90° left) (Supplementary Fig. 1c) for subsequent aesthetic ratings.

### Facial aesthetic ratings (FAR)

We recruited 10 experienced orthodontists as evaluators to give the facial aesthetics ratings (FAR). The FAR were on a scale of 0 to 10, where 0 represented the least attractive and 10 represented the most attractive. To avoid instrument and time-related systematic errors, the experts independently rated the photographs on the same screen simultaneously. For each participant, three FAR were obtained for frontal, profile, and overall views, respectively, at a specific time point (before and after orthodontic treatment). During the rating process, experts were not allowed to discuss with each other.

To maintain a natural appearance of facial aesthetic, experts were not given standardized training before the evaluation. They were only asked to assess the photos based on their own experience and subjective judgment. For each view of the face, we randomly included 5% (7 photographs) as duplicates for quality control purposes, to assess the consistency and reliability of the experts' ratings.

### Facial phenotype extraction based on photographic analysis

We defined the global facial phenotypes and clinical features based on the facial landmarks, specifically:

A total of 48 facial landmarks on frontal photographs were manually placed by experienced orthodontists according to the definition of traditional anthropometric measurements, including 35 facial soft tissue landmarks and 13 skeletal landmarks (Supplementary Fig. 2a, Supplementary Table 1). A total of 31 facial landmarks (24 soft tissue landmarks and 7 skeletal landmarks) were placed on right profile photographs (Supplementary Fig. 2b, Supplementary Table 1). For each photograph, coordinates for each landmark were acquired. Generalized Procrustes analysis (GPA) was then performed on the group of facial landmarks to eliminate any differences in position, orientation, and size of shapes, resulting in normalized facial landmarks.

Subsequently, we used two approaches to extract the facial phenotypes.

(1) Regarding the global facial phenotypes, we applied a dimensionality reduction approach by conducting Principal Component Analysis (PCA) on the coordinates of the facial landmarks for frontal and profile views separately. PCA was performed using:

$$X\approx {U}_{k}{\Sigma }_{k}{V}_{k}^{T}$$

where ${X}_{n\times p}$ is a matrix of normalized facial landmarks with *n* samples and *p* coordinates of landmarks, *k* is the number of retained principal components (PCs); ${\Sigma }_{k}$ is a diagonal matrix of the largest *k* singular values; and the column vectors of ${U}_{k}$ and ${V}_{k}^{T}$ are the corresponding *k* left and right singular vectors, where ${U}_{k}$ stand for principal components and ${V}_{k}^{T}$ stand for loadings. Here, we retained the first *k* = 15 principal components (${U}_{k}$) for further analysis to describe global facial shape variations associated with changes in facial aesthetics following orthodontic treatment.

(2) Regarding the clinical features, we extracted a total of 20 profile features and 17 frontal features, which were specifically selected based on their relevance to aesthetics or clinical orthodontic indicators. These features were derived from analyzing the proportions and angles of the facial landmarks (Supplementary Table 2). The purpose of extracting these features was to conduct a thorough analysis to determine which specific facial characteristics were correlated with aesthetic changes. By examining these selected features, we aimed to identify the key factors that contribute to changes in facial aesthetics following orthodontic treatment.

### Average face generation

The facial average face was constructed using the triangulation method. Initially, we applied an affine transformation to align each photograph, eliminating differences in position, size, and angle. Then, we calculated the average coordinates of the marked landmarks and performed Delaunay triangulation. For each triangle obtained by segmenting each photograph, we computed the affine transformation to map it to the corresponding triangle in the average shape. Finally, we calculated the pixel average of all photographs for each triangle, resulting in the generation of the average face.

### Data analysis

#### Association between FAR and global facial phenotypes

For both frontal and profile views, we performed multi-variate regression analysis to examine the association between the FAR and the facial principal components (${U}_{k}$) obtained through dimensional reduction of the facial landmarks. The multi-variate regression model can be expressed as follows:

$$FAR \sim {\beta }_{0}^{g}+\sum_{i=1}^{k}{\beta }_{i}^{g}{U}_{i}+{\varepsilon }_{g}$$

where ${\beta }_{i}^{g}$ denote the regression coefficients, which represent the influence of each facial principal component on the FAR; $\varepsilon$ stand for the error terms, capturing the unexplained variability in the FAR. The *P-values* of multivariate linear regression were calculated using the F-statistic (two-tailed), which quantifying the significance of the relationship between FAR and facial principal component features. Additionally, we defined weighted sums of PC loadings multiplied by regression coefficients as facial aesthetic vector (FAV) to quantify the changes in facial morphology corresponding to varying FAR, the formula can be expressed as follows:

FAV= {V}_{k}{\Sigma }_{k}{\beta }_{k}

### Association between FAR and clinical features

For each facial feature, we employed three methods to assess its influence on facial aesthetics.

- (1) Assuming that the average value had higher FAR [3, 4, 16], we performed regression analysis of the FAR with both the features and their squares to determine their correlation with FAR:

$$FAR \sim {\beta }_{0}^{c}+{\beta }_{1}^{c}c+{\beta }_{2}^{c}{c}^{2}+{\varepsilon }_{c}$$

where *c* denotes the particular clinical features, the *P-values* of were calculated using the F-statistic (two-tailed).

- (2) We used variance tests and T-test (two-tailed) to compare the changes in phenotype distributions before and after orthodontic treatment.
- (3) Using the absolute values of differences in FAR before and after orthodontic treatment and the corresponding absolute values of phenotype changes, we conducted regression analysis to assess whether orthodontic changes in a particular clinical feature were related to FAR, the *P-values* for ${\beta }_{1}$ were calculated using the T-statistic (two-tailed):
  $$\left|{FAR}_{after}-{FAR}_{before}\right|= {\beta }_{0}^{c}+{\beta }_{1}^{c}\left|{c}_{after}-{c}_{before}\right|+ {\varepsilon }_{c}$$

All the three tests above were conducted with a significance level of *P-value* < 0.01, and phenotypes showing significance in all three tests were considered more crucial in orthodontic treatment.

### Multifactor analysis of clinical features' impact on FAR

We employed multivariate regression methods to analyze the correlation between overall FAR and age, gender, and the three important clinical features identified using the test above. This step aimed to identify the most significant facial features related to aesthetic, the formula can be expressed as follows, the *P-values* for each clinical feature were calculated using T-statistic (two-tailed):

$$FAR={\beta }_{0}^{c}+\sum_{i=1}^{n=3}{\beta }_{i}^{c}{c}_{i}+ {\beta }_{age}{c}_{age}+{\beta }_{gender}{c}_{gender}+{\varepsilon }_{c}$$

## Results

### Characteristics of patients

A total of 73 patients were included in this study, including 18 males and 55 females, with an average age of 21 ± 8 years at the initial consultation. Further details are presented in Table 1.

**Table 1:** Patient information and orthodontic treatment measures

	Female	Male	Total
Malocclusion Type (Dental Diagnosis)			
I	16	3	19
II	21	7	28
III	18	8	26
Orthodontic Treatment Methods			
Labial	22	9	31
Invisible	11	4	15
Lingual	22	5	27
Premolar Extraction			
Yes	29	5	34
No	26	13	39
Orthognathic Surgery			
Yes	13	8	21
No	42	10	52
Bimaxillary Protrusion			
Yes	10	3	13
No	45	15	60
Mandibular Retrognathia			
Yes	26	6	32
No	29	12	41
Total	55	18	73

### The impact of orthodontic treatment on facial aesthetic rating (FAR) enhancement

#### The results of far given by experts

We recruited 10 clinical orthodontists to independently rate the facial aesthetics of the 73 samples in terms of frontal, profile, and overall views before and after orthodontic treatment. We initially assessed the reliability of expert self-assessments by utilizing duplicate photographs. The Pearson correlation coefficients for the two FAR ranged from 0.527 to 0.903 for each expert (Supplementary Fig. 3a). Additionally, we calculated the average of the absolute differences in FAR between the two ratings, which ranged from 0.381 to 0.905 for each expert (Supplementary Fig. 3b). These findings indicate a high level of consistency in the experts' rating standards and demonstrate the reliability of FAR given by experts.

**Fig. 1:**
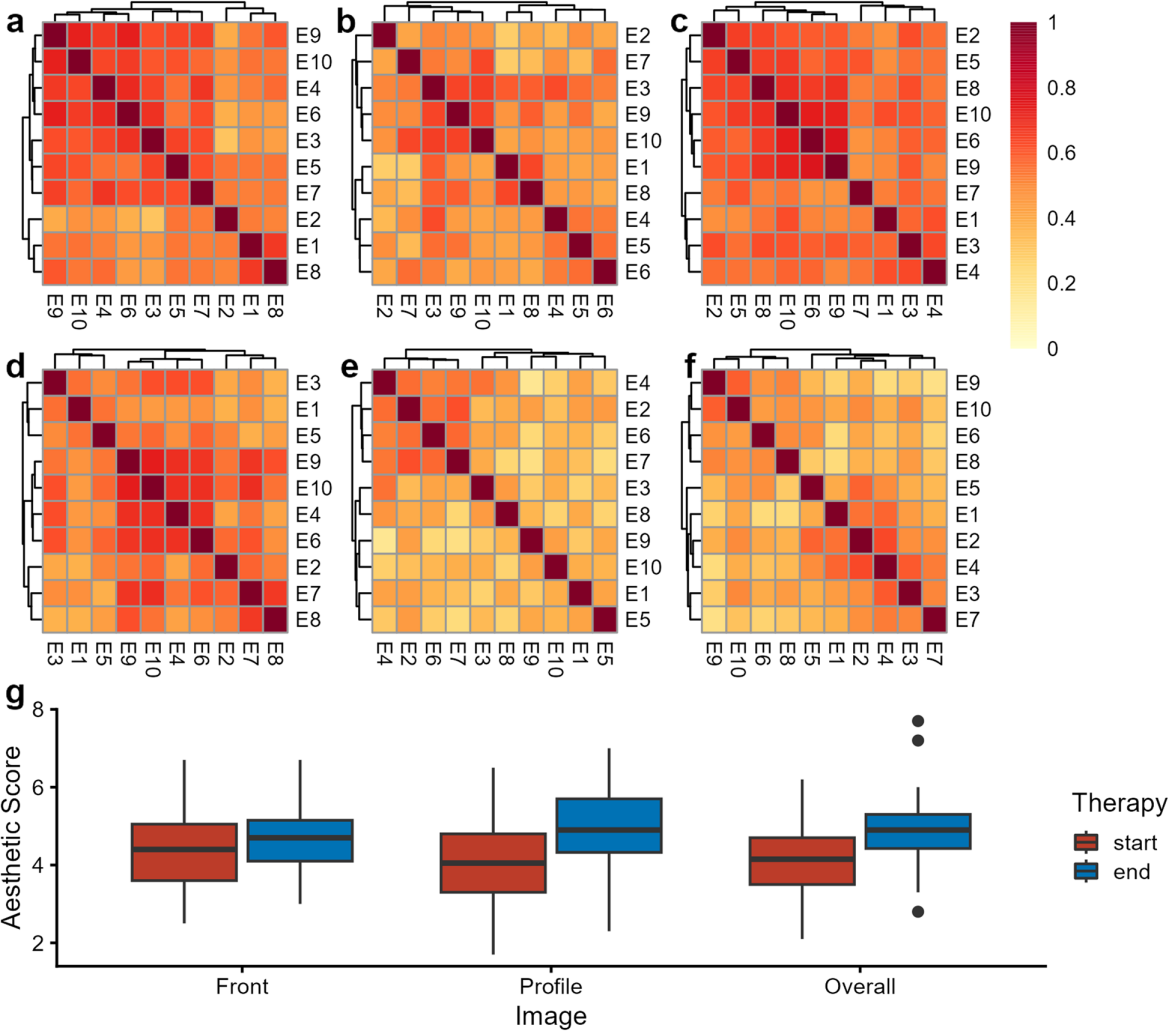
Consistency evaluation of far from experts. **a**-**f** Pearson correlation coefficient heatmaps of FAR among 10 experts before orthodontic treatment. Panels (**a**), (**b**), and (**c**) represent the correlations for frontal, profile, and overall FAR, respectively. Panels (**d**), (**e**), and (**f**) show the correlations for frontal, profile, and overall FAR, respectively, after orthodontic treatment. **g** Differences in frontal, profile, and overall FAR before and after orthodontic treatment

To analyze the consistency between the experts, we investigated whether the experts shared similar aesthetic evaluation standards between each other. We conducted Pearson correlation analyses for the FAR of frontal, profile, and overall views separately, both before and after orthodontic treatment (Fig. 1 a-f). The results revealed strong correlations among the FAR given by the experts in all six scenarios. Specifically, the consistency of FAR was higher for the frontal views before orthodontic treatment compared to after treatment. Moreover, the correlation in profile and overall views was higher than that for frontal views. Overall, the experts demonstrated relatively consistent rating criteria, particularly for profile views. Based on the consistency of FAR given by experts, we used the average FAR for subsequent analyses. The average FAR were presented in Table 2.

**Table 2:** FAR before and after orthodontic treatment

Photo Type	Sex	FAR before	FAR after	FAR difference	*P*-value
Frontal	Female	4.47	4.76	0.30	6.06 × 10^–3^
	Male	4.18	4.33	0.15	0.20
	All	4.40	4.66	0.26	4.00 × 10^–3^
Profile	Female	4.21	5.08	0.87	2.73 × 10^–8^
	Male	3.55	4.39	0.85	5.82 × 10^–3^
	All	4.05	4.91	0.86	1.03 × 10^–9^
Overall	Female	4.28	4.97	0.69	4.60 × 10^–7^
	Male	3.79	4.57	0.78	6.05 × 10^–3^
	All	4.16	4.87	0.71	1.64 × 10^–8^

*P*-value were of the FAR difference calculated using T-test

### The change of FAR before and after orthodontic treatment

Subsequently, we analyzed whether FAR improved after orthodontic treatment. We found that, regardless of frontal, profile, or overall FAR, the FAR were statistically higher after orthodontic treatment compared to before (Fig. 1g, Table 2). Among them, the improvement in profile and overall FAR were more substantial, with an average increase of 0.86 points for profile ratings (*P* = 1.03 × 10^–9^) and 0.71 points for overall ratings (*P* = 1.64 × 10^–8^). Frontal FAR showed a slight improvement, with an increase of 0.26 points (*P* = 0.004) (Table 2). These results indicated that the FAR showed enhancement after orthodontic treatment, demonstrating the effectiveness of orthodontics in improving facial aesthetics, especially in profile view.

### The influences of therapy to the change of FAR

**Fig. 2:**
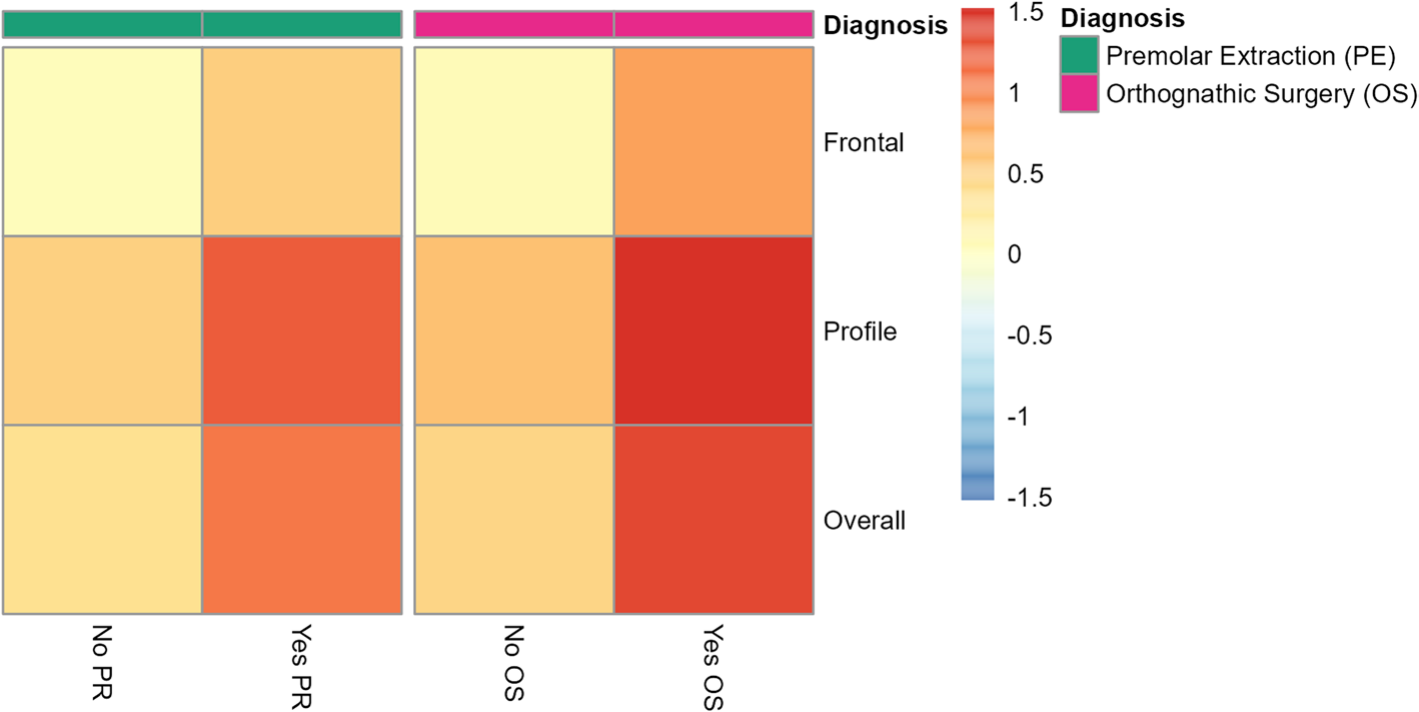
The impact of different orthodontic treatment measures on FAR

We also assessed the impact of premolar extraction and orthognathic surgery on FAR before and after orthodontic treatment. We found that patients who received premolar extraction showed a higher FAR compared to those who did not undergo extraction (Fig. 2, Supplementary Table 3). Similarly, patients who underwent orthognathic surgery also experienced substantial changes in FAR before and after orthodontic treatment (Fig. 2, Supplmentary Table 3). Regarding the orthodontic treatment methods, lingual treatment showed the highest improvement, while invisible treatment showed the least improvement. Additionally, the effectiveness of aesthetic enhancement after orthodontic treatment did not vary significantly among patients with different clinical conditions, such as mandibular retrognathia and different malocclusion types (Supplementary Table 3).

Colors represent the changes in FAR.

### The change of global facial morphology from orthodontic treatment and FAR improvement

#### The change of global facial morphology after orthodontic treatment

**Fig. 3:**
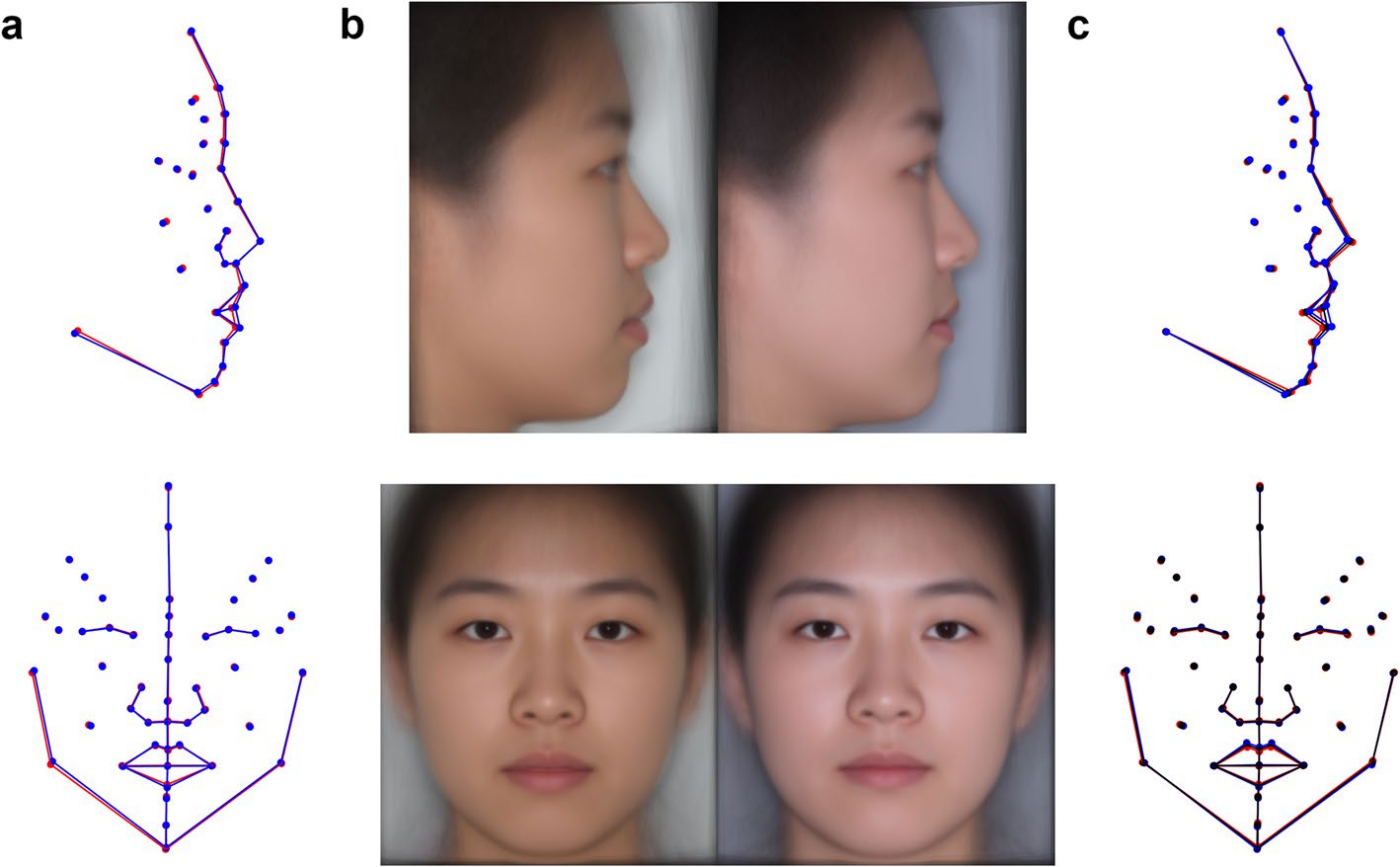
The correlation of global facial phenotype with FAR and orthodontic treatment. **a** Changes in facial morphology before and after orthodontic treatment for profile view (top) and frontal view (bottom). (Blue represents the average facial shape before orthodontic treatment, and red represents the average facial shape after orthodontic treatment.) **b** Comparison of average facial shape before and after orthodontic treatment for profile view (top) and frontal view (bottom). **c** Association between facial morphology changes and FAR for profile view (top) and frontal view (bottom). (Black represents the facial average shape, red represents facial shapes with higher FAR, and blue represents facial shapes with lower FAR.)

We compared the positional changes of facial landmarks before and after orthodontic treatment and found that the changes in facial morphology after orthodontic treatment were mainly concentrated in the lower third of the face (Fig. 3a). For the profile view, the main changes involved inward displacement of the ls, sto, and li points, indicating a more retracted position of the lips. The sm point showed a slight inward movement, while the pg and gn points showed a minor outward displacement, representing a slightly protruding chin. For the frontal view, the main change was the reduction in lip height, indicating thinner lips. Changes were observed in the vertical direction of the ls and li points.

We performed T-tests on the lateral and vertical components of each facial landmark before and after orthodontic treatment. Similarly, statistically significant changes were observed in the lateral components of the ls, sto, and li, all showing a more inward position (P_ls.x_ = 1.43 × 10^–5^, P_sto.x_ = 1.92 × 10^–8^, P_li.x_ = 3.86 × 10^–9^, one-tail). In the vertical components, the ls point showed a downward movement (P_ls.y_ = 1.70 × 10^–7^, one-tail), resulting in a reduction in lip thickness.

We also generated average facial images before and after orthodontic treatment (Fig. 3b). By observing the average face images, we observed obvious changes in the mouth area, which were consistent with the results obtained based on facial landmarks. Besides, the facial changes in frontal (Supplementary Fig. 4) and profile (Supplementary Fig. 5) views after orthodontic treatment were similar for both males and females. And the degree of changes was measured using the Procrustes distance, showing no significant differences between two sexes (P_frontal_ = 0.80, P_profile_ = 0.34).

### The associations between FAR and the global facial phenotypes

We used Principal Component Analysis (PCA) to perform dimensionality reduction on the facial landmark coordinates and kept the first 15 principal components (PCs) as global facial phenotypes. Then, we conducted multivariate linear regression between the FAR and these 15 PCs. We showed statistically significant association between FAR and global facial phenotypes (P_frontal_ = 3.13 × 10^–9^; P_profile_ < 2.2 × 10^–16^). We visualized the most relevant facial aesthetic vectors (FAV) representing changes in facial morphology when FAR improved from low to high. For the profile view, the most important changes as FAR increased were mainly in the lip area, characterized by inward retraction of the lips and slight chin protrusion (Fig. 3c). These changes were also mainly in the ls, sto, li, sm, and pg facial landmarks. These changes in facial morphology were consistent with the average facial morphology changes before and after orthodontic treatment, confirming that the improvement in facial aesthetics from orthodontic treatment was associated with the inward retraction of the lips in the profile view. For the frontal view, the most relevant facial changes associated with FAR were also in the lip area, as lip height decreased.

In summary, the impact of orthodontic treatment on facial morphology primarily focused on the lip and chin regions. Lip changes were characterized by inward retraction and reduced height, while chin changes exhibited outward protrusion and increased fullness. These changes were consistent with the direction of improvement in FAR, demonstrating the effectiveness of orthodontic treatment in enhancing facial aesthetics for both frontal, profile, and overall views.

### The clinical features increased FAR through orthodontic treatment

Although the global facial phenotype could catch the overall changes with increasing FAR, specific recommendations cannot be provided to clinical practice due to the lack of specific measurement indicators. Therefore, we selected 37 clinical features based on angles and proportions between facial landmarks (20 in profile view and 17 in frontal view) that were previously reported to be associated with facial aesthetics or orthodontic clinical indicators (Supplementary Table 2), to identify which features contribute to improving FAR in orthodontic treatment.

Specifically, we conducted three tests to identify the key clinical features in orthognathic surgery: 1) We employed quadratic regression analysis to assess the correlation between FAR and clinical features for all samples, to identify which features were related to facial aesthetics. 2) We tested the variance (var-test) and mean (T-test) of feature distributions before and after orthodontic treatment, to determine which features changes after orthodontic treatment. 3) We examined the correlation between the absolute differences in FAR before and after orthodontic treatment and the absolute differences in clinical features, to identify which feature changes are related to facial aesthetics. These tests aimed to identify the clinical features which are key to the orthodontic treatment.

### Correlation between profile clinical features and FAR

For the profile clinical features, we analyzed the correlations between the profile and overall FAR separately. We found nine features changes after orthodontic treatment; four features were associated with FAR; three feature changes were associated with FAR changes (Supplementary Table 4).

**Fig. 4:**
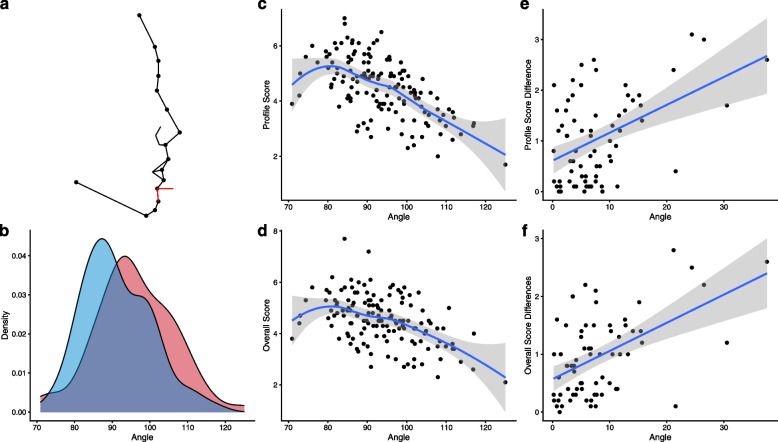
The association of pg.sm.hori with orthodontic treatment and FAR. **a** Schematic representation of the feature, with the pg.sm.hori angle indicated by a red line. **b** Density plots of the feature values before (in red) and after (in blue) orthodontic treatment. **c**, **d** The correlation between the feature values and **c** profile and **d** overall FAR. **e**, **f** The correlation between the absolute difference in feature values before and after orthodontic treatment and **e** the absolute difference in profile and **f** overall FAR

**Fig. 5:**
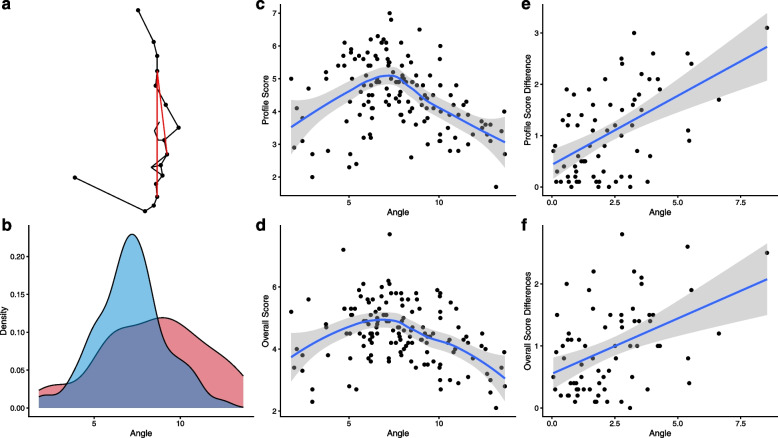
The association of pg.n.ls with orthodontic treatment and FAR. **a** Schematic representation of the feature, with the pg.n.ls angle indicated by a red line. **b** Density plots of the feature values before (in red) and after (in blue) orthodontic treatment. **c**, **d** The correlation between the feature values and **c** profile and **d** overall FAR. **e**, **f** The correlation between the absolute difference in feature values before and after orthodontic treatment and **e** the absolute difference in profile and **f** overall FAR

Among these facial features, pg.sm.hori and pg.n.ls showed statistically significant correlations in all three tests, suggesting that these two features were closely related to FAR and underwent statistically significant changes through orthodontic treatment, leading to an improvement in FAR. Specifically, pg.sm.hori achieved the highest FAR at around 80°, and after orthodontic treatment, the mean value decreased by 4°, approaching the point of the highest FAR. This indicated a statistically significant impact of orthodontic treatment on this feature, resulting in an increase in FAR (Fig. 4). Similarly, for pg.n.ls, the highest FAR was achieved at around 8°, and after orthodontic treatment, the mean value approached the point of the highest FAR, demonstrating the influence of orthodontic treatment on this feature and its contribution to the improvement in FAR (Fig. 5).

### Correlation between frontal clinical features and FAR

For the frontal facial features, we analyzed the correlations between the frontal, and overall FAR separately. We found that three features’ changes after orthodontic treatment; three features are associated with FAR; two feature changes were associated with FAR changes (Supplementary Table 5). But we did not find any features that were statistically significant in all three tests. But we found that sto-sm/sn-gn were associated with FAR, and the feature changed after orthodontic treatment (Supplementary Fig. 6). Overall, the changes in frontal facial features were not as significant as those observed in the profile view.

### Multifactor analysis of the correlation between clinical features and overall FAR

We aimed to explore the most important clinical features in orthodontic treatment to facial aesthetic. Using the difference in overall FAR before and after orthodontic treatment as the dependent variable, and age, gender, and three clinical features identified above (pg.sm.hori, pg.n.ls, and sto-sm/sn-gn) as independent variables, we conducted a multifactor analysis. We found that pg.sm.hori remained statistically significant (*P* = 0.0014), indicating that this facial feature may require special attention during orthodontic treatment (Supplementary Table 6).

## Discussion

Orthodontics is the conventional treatment for dental and facial malocclusions, usually focusing more on changes in the profile and lower third of the face. In this study we focused on the quantified changes in facial aesthetic induced by orthodontic treatment. By collecting pre- and post-orthodontic facial photographs from a clinical cohort, facial features were systematically extracted using based on facial landmarks, and in combination with FAR given by experts, the impact of orthodontics on facial aesthetics was analyzed. The findings not only underscore the substantial impact of orthodontic treatments, particularly from profile perspectives, but also emphasize the pivotal role of specific clinical features like pg.sm.hori and pg.n.ls in facial aesthetics. These findings could help orthodontists to better understand and optimize the aesthetic outcomes of orthodontic treatment.

Our study utilized two types of phenotyping approaches based on facial landmarks. The global facial phenotypes involved all landmarks, which give a whole facial change with FAR increase. This allows us to receive intuitive feeling of the most important facial area with facial aesthetic. Here is lip area in both profile and frontal view. However, because lack of specific indicator, this approach is difficult to applicate to clinical practice. Thus, we collected 37 clinical features, and used three test to identify the key features which are both associated with facial aesthetic and orthodontic treatment. In results, pg.sm.hori and pg.n.ls were found to be the key factors. Although these two phenotyping approaches were designed for different purposes, their results were consistent and mutually supported each other, reinforcing their significance in understanding the relationship between orthodontics and facial aesthetics.

Our results showed that patients' FAR improved after orthodontic treatment, whether in profile, frontal, or overall view. We observed that orthodontic treatment led to more pronounced changes in the profile aspects, primarily in the lip and mandibular regions. After orthodontics, facial morphology changes included lip retraction and increased mandibular fullness. Conversely, orthodontic changes in the frontal aspect were relatively minor, but the main alterations also focused on the lip region, mainly manifesting as a slight reduction in lip height, consistent with previous studies and with the current clinical investigation [17]. One possible explanation is that in our sample, the majority of patients have relatively symmetrical facial features. Further study is needed to explore the improvement of frontal facial aesthetics in other specific patients (such as those with laterognathia) undergoing orthodontic treatment. By considering tooth extraction as an evaluation factor, we discovered that the improvement in FAR was statistically significantly higher in the extraction group compared to the non-extraction group. Recent reflections on extraction orthodontics and concerns about "tooth extraction face" have led to increased patient anxiety [18–20]. Our results show that tooth extraction is beneficial for the overall aesthetic evaluation of patients, consistent with previous research findings [21]. Regarding of orthodontic treatment methods, although we observed greater aesthetic improvement in patients with lingual appliances, this may be attributed to that the majority of these patients underwent premolar extraction treatment (22/27, 81.5%), while fewer patients underwent premolar extraction with labial appliances (19/31) and invisible aligners (0/15). Further study with a larger sample size is needed to determine if there are any differences in aesthetic improvement among different types of orthodontic methods under similar conditions.

We summarized and analyzed previously reported orthodontic clinical features that are associated with facial aesthetics. Overall, similarly to the above conclusions, profile clinical features showed more statistically significant correlations with facial aesthetics in orthodontic patients. Furthermore, for profile aspects, although numerous metrics, including the upper and lower lip to E-line [22], facial convexity [23], have been correlated with aesthetic scores, only the phenotypes with strong correlations with both orthodontics and aesthetics were pg.sm.hori and pg.n.ls. These two phenotypes underwent statistically significant changes before and after orthodontics and were correlated with FAR. We found that the highest FAR for pg.n.ls was around 8°, which differs from previous research suggesting the optimal angle is 5.9° [24]. We attribute this difference to variations in the patient population and suggest that the reference standard for this angle can be set within a range, rather than being a specific value. For frontal aspects, most phenotypes related to aesthetic scores underwent minimal changes before and after orthodontics. Specifically, sto.sm/sn.gn experienced changes and was correlated with FAR, indicating that the proportion of the lower lip and lower third of the face is a crucial factor in frontal aesthetics, consistent with previous literature [25, 26]. Besides, clinical features like sto-sm/sn-gn, puR-puL/ex-en, alR-alL/ex-en et al., which are related to eye and nose, have a larger correlation with frontal FAR [27, 28]. These features did not have a statistically significant change after orthodontic treatment, suggesting that clinical features more related to frontal facial aesthetics may not be improved through orthodontics. Other approaches such as plastic surgery may be needed to improve frontal facial aesthetics.

Moreover, our study found that there was a strong consensus among experts in assessing facial aesthetics, with the highest consistency observed in profile views, suggesting that orthodontists may share more consistent opinions on facial attractiveness in profile aspects due to systematic and traditional training as well as aesthetic perception education [29]. However, the consistency in frontal views was lower, indicating that there may be more individual factors affecting aesthetic evaluations in the frontal view, such as the evaluator's gender, age, occupation, and preferences [30, 31].

In addition, this study has some limitations. Firstly, being a retrospective study, the sample size is limited, and lack statistical power to detect some previously reported features related to orthodontic aesthetics. We have initiated a prospective study that included a larger sample size and grouped patients according to type of malocclusion, gender and age for more comprehensive research that provides a comprehensive picture of the relationship between orthodontics and facial aesthetics. Additionally, this study only recruited orthodontists for FAR, therefore, the facial aesthetic assessments in this study only could represent orthodontists. Some studies have indicated differences in aesthetic judgment criteria for facial aesthetics among orthodontists, patients, and the general population [32, 33]. It would be informative to include ratings from plastic surgeons, patients, and the general individuals to investigate whether there are similar or different aesthetic perception among different groups. Furthermore, with the advancements and increasing availability of 3D imaging technologies, future studies could utilize 3D facial scans to obtain more precise data about the true facial morphology of subjects, and to fabricate customized appliances for patients who have undergone orthodontic treatments, which could be used to improve both the functional and aesthetic outcomes of treatment [34].

## Conclusion

In conclusion, this study utilized facial photographs of orthodontic patients and expert ratings to systematically investigate the impact of orthodontics on facial aesthetics. The results demonstrated that orthodontic treatment generally improves facial aesthetic, especially for profile aspect showing a larger improvement. Moreover, we explored the relationship between clinical features and facial aesthetics, discovering that profile features, especially pg.sm.hori and pg.n.ls, had the most significant correlations with facial aesthetics. These two phenotypes underwent substantial changes after orthodontics and were correlated with FAR. These findings provide essential references and guidance for the clinical application of orthodontic treatment and facial aesthetics research. While there are limitations to the study, by expanding the sample size, considering ratings from patients and the general population, and utilizing 3D facial scanning, we can further investigate the relationship between orthodontics and facial aesthetics, contributing to the development of facial aesthetics and enhancing the quality of life for individuals.

### Supplementary Information

**Additional file 1. **Supplementary Tables.

**Additional file 2: Supplementary Figure 1. **Reference Photos for Expert Ratings.** Supplementary Figure 2. **Illustration of Facial Soft Tissue Landmarks (in red) and Skeletal Landmarks (in green).** Supplementary Figure 3. **The replication of FAR given by orthodontic experts.** Supplementary Figure 4. **Comparison of the frontal view before and after orthodontic treatment in males and females.** Supplementary Figure 5. **Comparison of the profile view before and after orthodontic treatment in males and females. **Supplementary Figure 6. **The Association of sto-sm/sn-gn with Orthodontic Treatment and FAR.

## Data Availability

The data that support the findings of this study are available from Shanghai Ninth People's Hospital, Shanghai Jiao Tong University School of Medicine but restrictions apply to the availability of these data, which were used under license for the current study, and so are not publicly available. Additional details can be requested from Bing Fang (fangbing@sjtu.edu.cn).

## Abbreviations

FAR: Facial Aesthetic Ratings
PC: Principal Components
PCA: Principal Component Analysis
