# Supplementary material for: Impact of orthodontic-induced facial morphology changes on aesthetic evaluation: a retrospective study
Source: BMC Oral Health. 2024 Jan 5;24:24. doi: 10.1186/s12903-023-03776-4 (PMC10768126; doi:10.1186/s12903-023-03776-4)
Supplement: Supplementary file 2 — Additional file 2: Supplementary Figure 1. Reference Photos for Expert Ratings. Supplementary Figure 2. Illustration of Facial Soft Tissue Landmarks (in red) and Skeletal Landmarks (in green). Supplementary Figure 3. The replication of FAR given by orthodontic experts. Supplementary Figure 4. Comparison of the frontal view before and after orthodontic treatment in males and females. Supplementary Figure 5. Comparison of the profile view before and after orthodontic treatment in males and females. Supplementary Figure 6. The Association of sto-sm/sn-gn with Orthodontic Treatment and FAR. [file 12903_2023_3776_MOESM2_ESM.docx]

**Supplementary Figure**


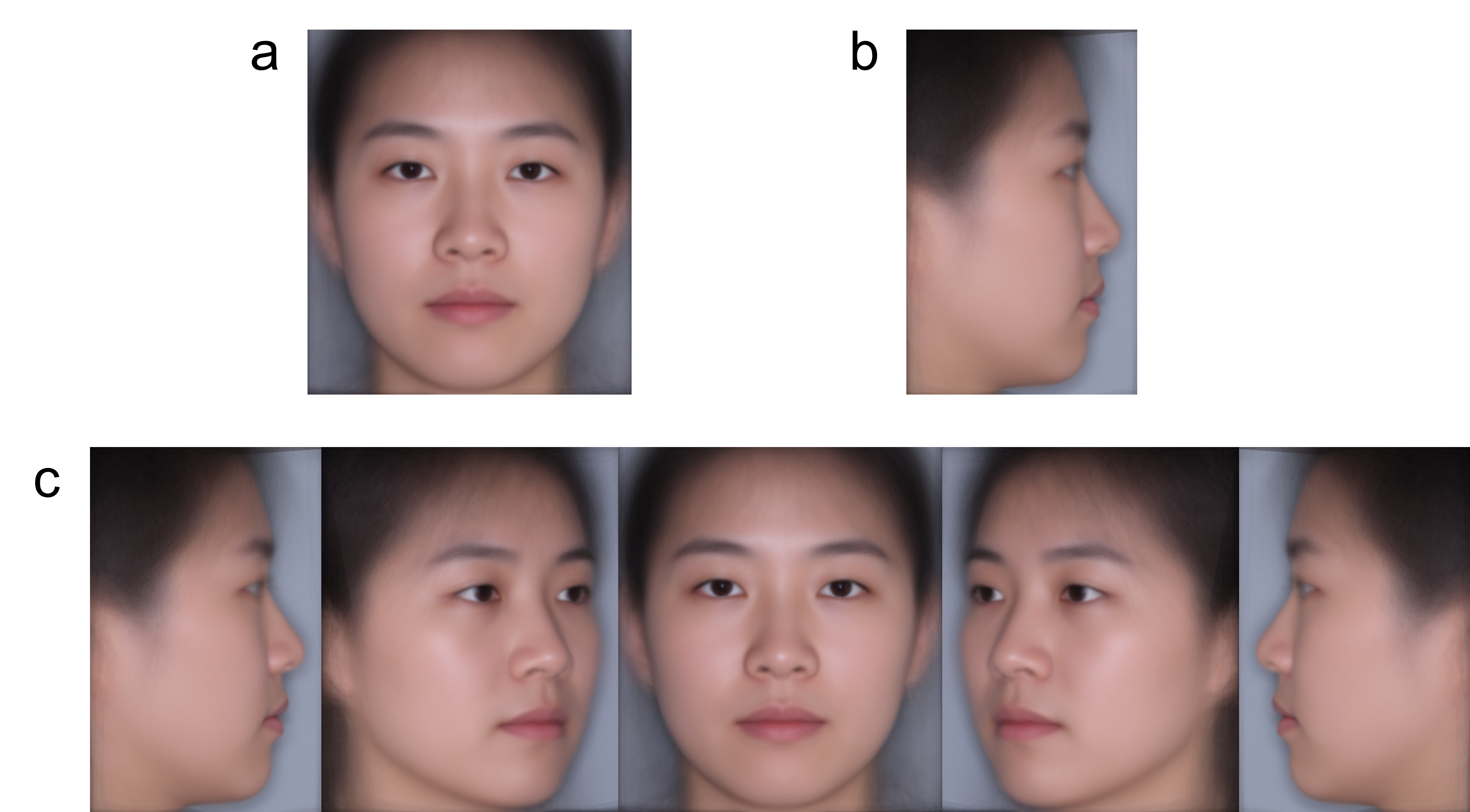
**Supplementary Figure 1: Reference Photos for Expert Ratings.**

a: Frontal View Photo; b: 90-degree Profile View Photo; c: Overall Photos. The average faces were used for reference photos.


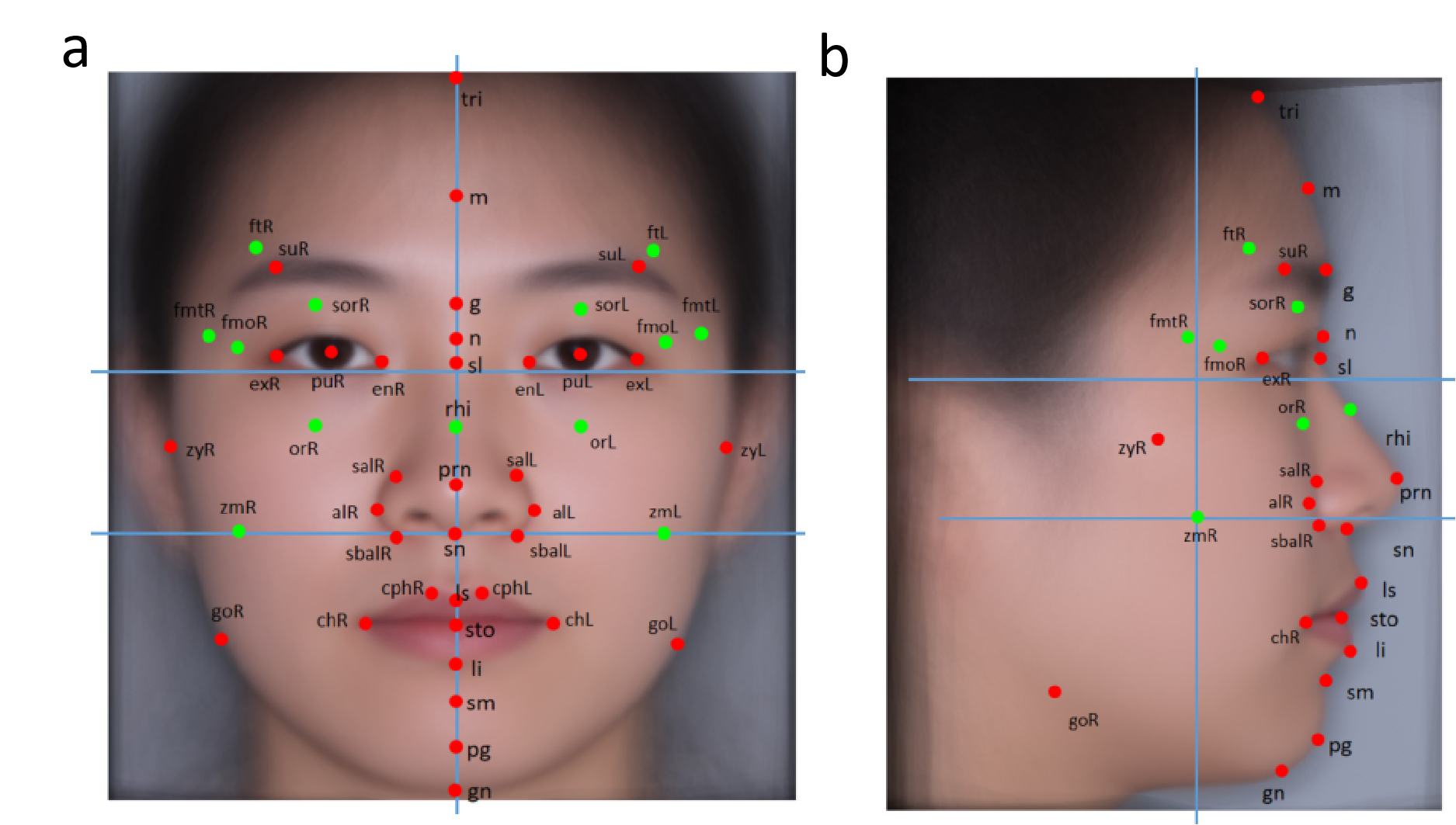


**Supplementary Figure 2: Illustration of Facial Soft Tissue Landmarks (in red) and Skeletal Landmarks (in green).**

a: Frontal View Photo; b: 90-degree Lateral View Photo. The average faces were used for reference photos.


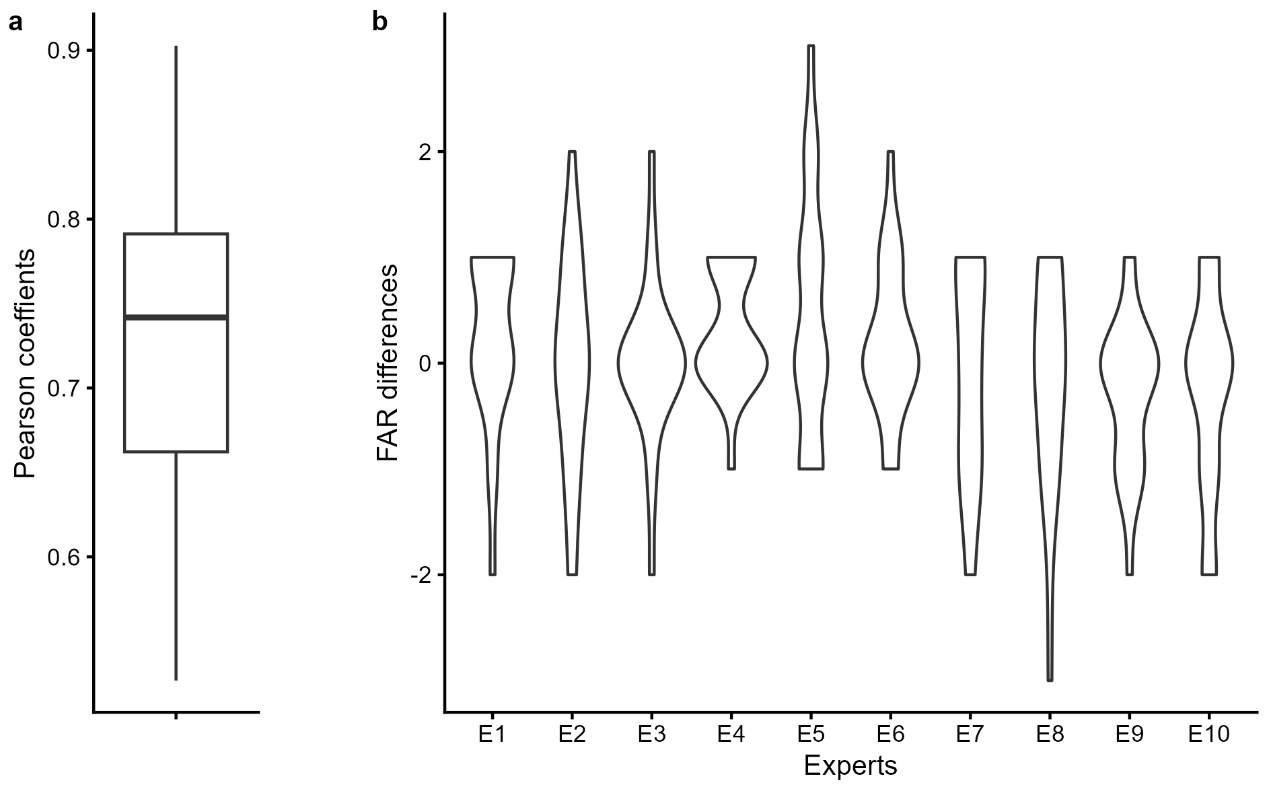


**Supplementary Figure 3: The replication of FAR given by orthodontic experts**

a: Boxplot of Pearson correlation coefficients for the two FAR of 21 repeated photos from 10 experts; The FAR differences for 21 repeated photos of 10 experts.


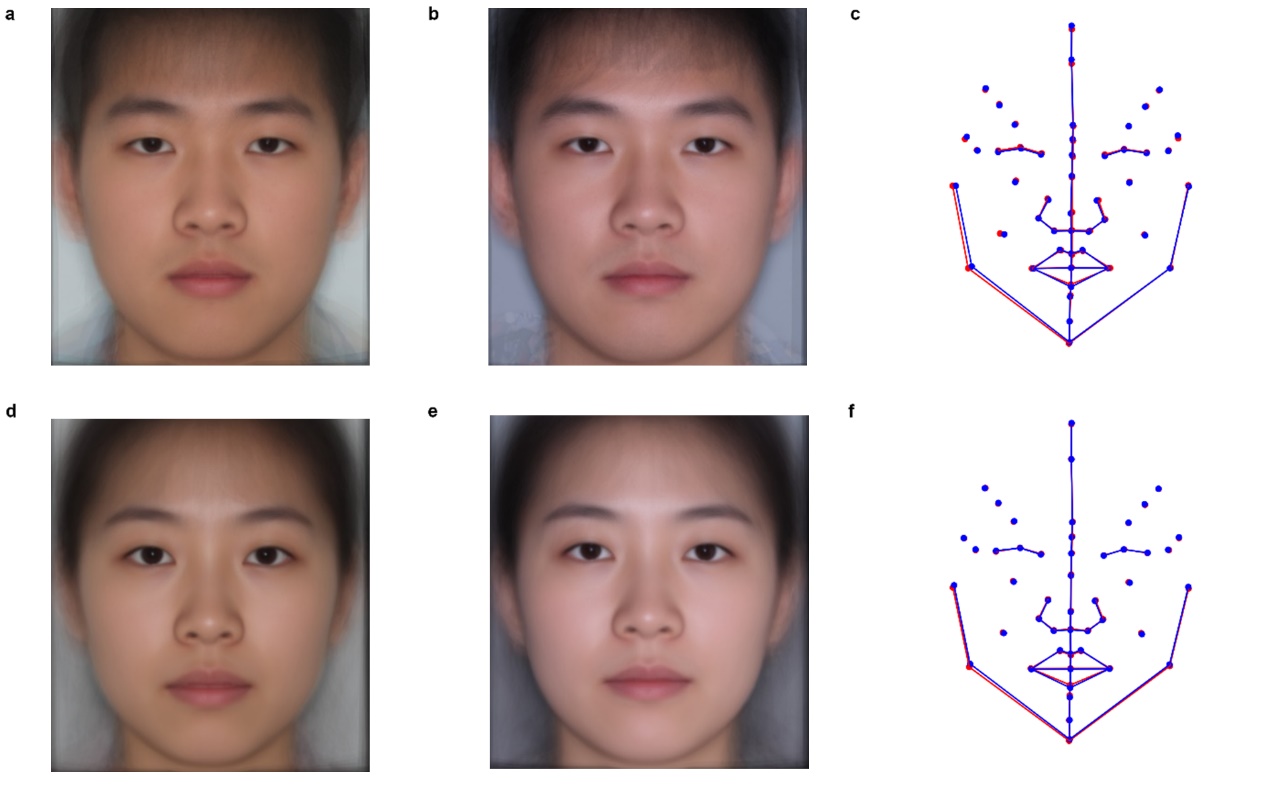


**Supplementary Figure 4: Comparison of the frontal view before and after orthodontic treatment in males and females.**

Average face of males a) before and b) after orthodontic treatment; c) Changes in facial morphology before and after orthodontic treatment in males; Average face of females d) before and e) after orthodontic treatment; f) Changes in facial morphology before and after orthodontic treatment in females (Blue and red represents the average facial shape before and after orthodontic treatment separately)


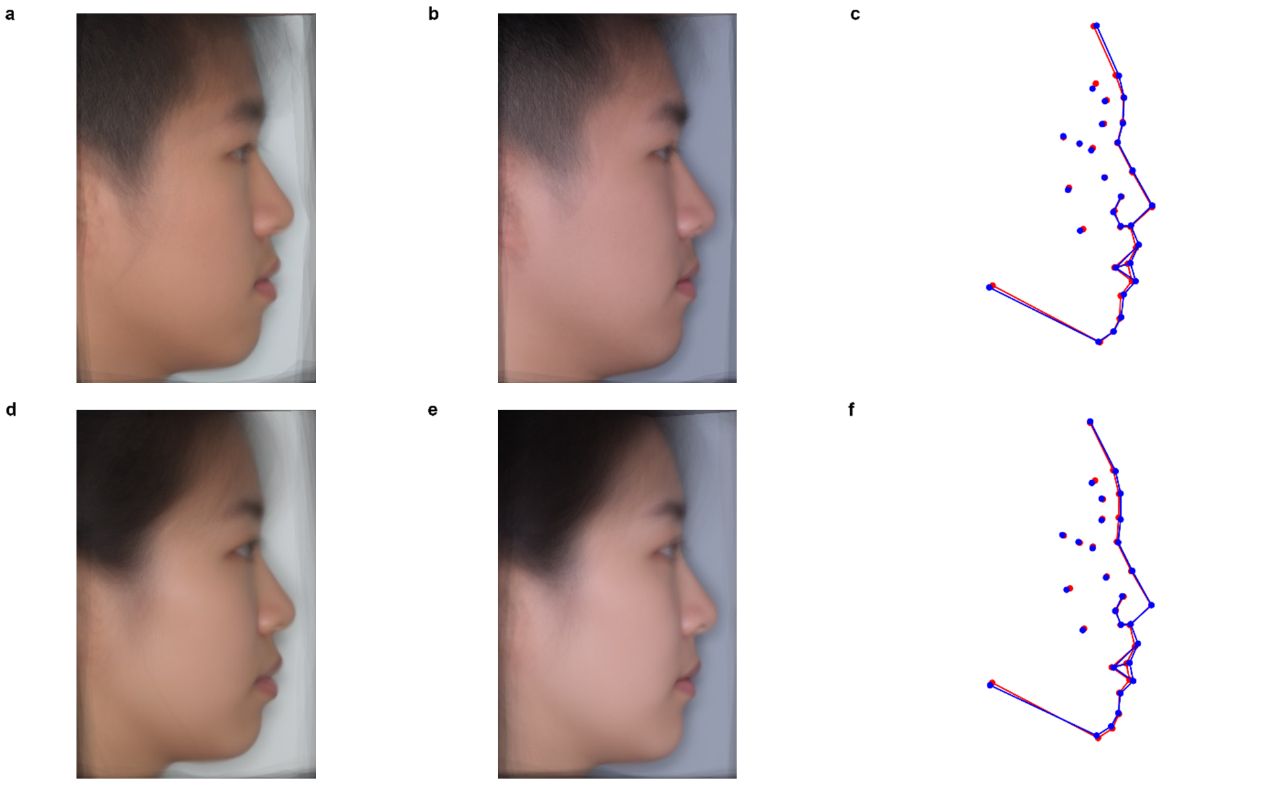


**Supplementary Figure 5: Comparison of the profile view before and after orthodontic treatment in males and females.**

Average face of males a) before and b) after orthodontic treatment; c) Changes in facial morphology before and after orthodontic treatment in males; Average face of females d) before and e) after orthodontic treatment; f) Changes in facial morphology before and after orthodontic treatment in females (Blue and red represents the average facial shape before and after orthodontic treatment separately)


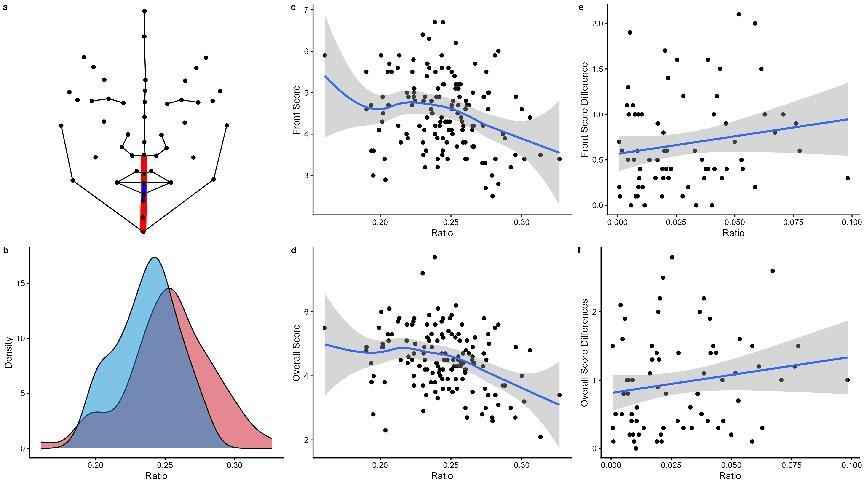


**Supplementary Figure 6: The Association of sto-sm/sn-gn with Orthodontic Treatment and FAR**

a) Schematic representation of the features, with sto-sm/sn-gn indicated by a red line. b) Density plots of the feature values before (in red) and after (in blue) orthodontic treatment. c, d) The correlation between the feature values and c) frontal and d) overall FAR. e, f) The correlation between the absolute difference in feature values before and after orthodontic treatment and e) the absolute difference in frontal and f) overall FAR.
